# Characterization of in-barn heat processed swine mortalities

**DOI:** 10.3389/fvets.2023.929160

**Published:** 2023-03-20

**Authors:** Brett C. Ramirez, Ryan Jeon, Dave R. Stender, Kris D. Kohl, Chris J. Rademacher, Justin T. Brown, Dwight Mogler

**Affiliations:** ^1^Department of Agricultural and Biosystems Engineering, Iowa State University, Ames, IA, United States; ^2^Extension and Outreach, Iowa State University, Ames, IA, United States; ^3^Department of Veterinary Diagnostic and Production Animal Medicine, Iowa State University, Ames, IA, United States; ^4^Mogler Farms/Pig Hill Co., Lester, IA, United States

**Keywords:** foreign animal disease, temperature, carcass, response, disposal, management

## Abstract

In-barn heat processing of mass swine mortalities to inactivate pathogens could facilitate more carcass disposal options and reduce the risk of pathogen spread in the event of a foreign animal disease (FAD) outbreak. A 12.2 × 12.2 × 2.4 m (W × L × H) heat processing room was created using a temporary wall inside a de-commissioned commercial gestation barn in northwest Iowa. Eighteen swine carcasses (six per group) divided into three weight groups (mean ± SD initial carcass weights: 31.8 ± 3.3, 102.7 ± 8.1, and 226.3 ± 27.6 kg) were randomly assigned a location inside the room. Three carcasses per weight group were placed directly on concrete slats and on a raised platform. One carcass per weight group and placement (n=6) was instrumented with five temperature sensors, inserted into the brain, pleura, peritoneal, ham, and bone marrow of the femur, and a sensor was attached directly to the skin surface. Environmental conditions (ambient and room) and carcass temperatures were collected at 15-min intervals. Carcasses were subjected to an average room temperature of 57.3 ± 1.2°C for 14 days. The average (±SD) reduction from initial weight for the carcasses on slats was 45.0 ± 4.70% (feeder), 33.0 ± 8.30% (market), and 34.0 ± 15.80% (sow), and for the carcasses on a raised platform, it was 39.0 ± 6.80% (feeder), 49.0 ± 11.30% (market), and 45.0 ± 6.70% (sow). There was a significant interaction between carcass placement (slats and raised) and carcass weight loss for the market weight group. When average carcass surface temperature was at 40.6, 43.3, and 46.1°C (data grouped for analysis), the average internal carcass temperature for most measurement locations was significantly different across carcass weight groups and between the carcasses on a raised platform and those on slats. This preliminary analysis of carcass weight loss, leachate production, and temperature variation in carcasses of different sizes can be used for planning and evaluating mass swine mortality management strategies.

## Introduction

A foreign animal disease (FAD) outbreak would cause widespread devastation across the US swine industry. African Swine Fever (ASF) is a highly resilient virus of paramount relevance that causes high morbidity and mortality (1). By February 2019, an estimated 45M pigs had been culled in China due to ASF, with an economic loss of nearly $8.5B [estimated at 100 kg hd^−1^ at 13.5 RMB kg^−1^; (2)]. Comparatively, a FAD outbreak in the US could cost upwards of $50B over 10 years (3). While extreme prevention measures can be deployed and enforced, a confirmed ASF outbreak would force an instant stop movement response to limit the viral spread, locate infected premises, eradicate the virus, and dispose of infected mortalities. Mortality management of large-scale, infected carcasses is of utmost concern because it directly impacts the spreading and potential eradication of the virus. Current mass mortality management approaches for swine include composting, shallow burial, landfill disposal, rendering, and incineration (4). All these methods require the removal of infected carcasses from the site and the exposure of the people and equipment involved in the disposal process. Since the ASF virus is very resilient in the environment, this creates an issue wherein the virus could be transmitted during the disposal process and potentially infect the equipment, which could serve as a vector to potentially infect other naïve pig sites and perpetuate the outbreak. If the pathogen could be inactivated prior to the carcass removal and disposal process, there would be a reduced risk of exposure of infected mortalities or leachate.

Due to the biosecurity challenges associated with existing management strategies for mass swine mortalities, the inactivation of pathogens prior to removal from the facility could enhance many disposal options. During catastrophic poultry mortality events, in-barn mortality management strategies have been tested and deployed with success (5, 6). Approximately half the labor is needed to manage poultry carcasses in-barn compared to traditional carcass disposal methods, thereby reducing disease transmission risk by workers (7). Further, the cost is relatively low (i.e., minimal extra materials or equipment needed) and elevated temperatures (e.g., 60°C for 20 min of ASF inactivation) are easily generated and sustained with existing heaters, decreasing the transmission of pathogens to the surrounding environment (7). Therefore, in-barn mortality management strategies for swine warrants investigation on a commercial scale to determine the feasibility of heat-treating swine carcasses in the barn.

The overall goal of this work is to establish an initial understanding of the feasibility of viral thermal inactivation inside swine carcasses and buildings with minimal additional personnel/supplies. If effective, which is assessed by the achievement of proven time and temperature relationships, the number of disposal options could be increased due to the reduced threat of moving infected carcasses and leachate off-site. Since carcasses will be exposed to elevated temperatures achieved by forced air heat processing over an extended period of time, carcass weights should be reduced, thereby decreasing total weight for disposal. This project is the first to preliminarily study temperature distribution throughout a test section of a commercial-scale swine facility subjected to elevated and constant temperatures achieved *via* a heat treatment process. The objectives of this preliminary study are to (1) characterize environmental parameters during in-barn heat processing; (2) assess carcass thermal responses; and (3) assess total carcass weight loss.

## Materials and methods

### Facility description

**Figure 1:**
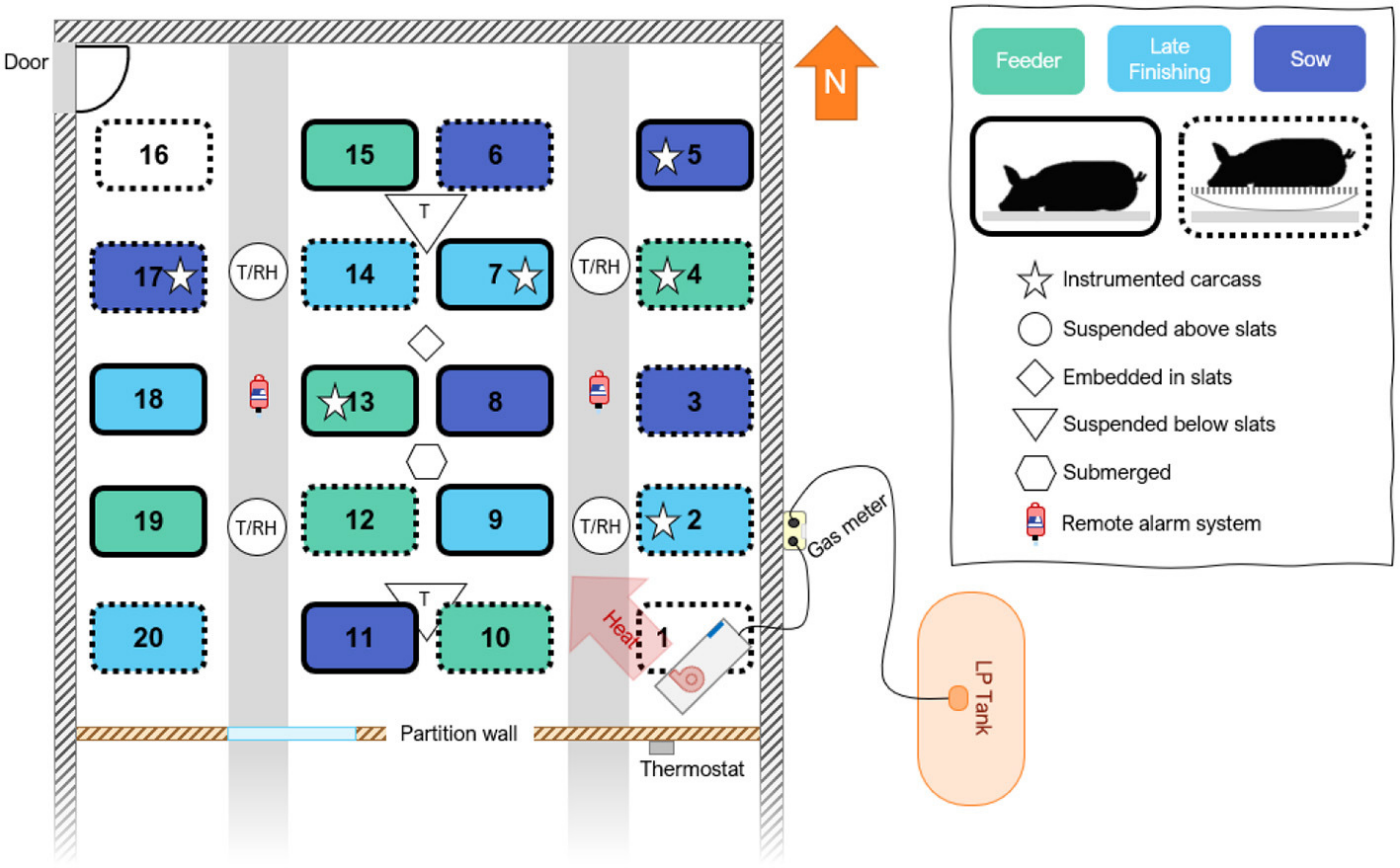
Layout of test room indicating sensor locations, heater, and carcass placement. Six pigs representing three weight ranges were placed directly on the slats (solid outline) and six pigs were placed on a metal platform raised above the slats to collect leachate.

The preliminary study took place in a decommissioned swine gestation building (nominally: 36.6 × 12.2 × 2.4 m; L × W × H) with solid-sided (insulated) walls, located in northwest Iowa with partially slatted concrete slats and two raised fully concrete aisles. A temporary wall was constructed inside the building, with wood framing, rigid Styrofoam insulation, and a vapor barrier to create a room approximately one-third of the size of the original space. This was performed to reduce the room size for heating while preserving the features of a commercial-scale facility. The test room created in the north end of the building had interior dimensions of 12.2 (L) by 12.2 (W) by 2.4 (H) m (Figure 1). A 2.4 m deep manure pit was approximately half full of predominantly water (slurry and solids were previously removed) throughout the experiment. One door located in the northwest corner allowed entry and exit from the room. The flooring was marked with 20 locations; four rows by five columns were evenly distributed throughout the test section to indicate placement positions for the carcasses.

Inside the room, a direct gas-fired circulating heater (73.3 kW; 250,000 BTU h^−1^; Guardian 250, L.B. White Company, Onalaska, WI, USA) was placed in the southeast corner and was controlled by a weatherproof digital thermostat (TSW-150, Dwyer Instruments Inc., Michigan City, IN, USA) with a temperature sensor located inside the test section (Figure 1). A 3.78 m^3^ (1,000 gallons) liquid propane (LP) tank located adjacent to the building supplied fuel to the heater.

### Instrumentation

Ambient conditions were monitored with a temperature/relative humidity (±0.20°C from 0 to 70°C; ±2.5% from 10 to 90% RH; S-THC-M002, Onset Computer Corporation, Bourne, MA, USA) sensor placed inside a passive solar radiation shield (RS3-B, Onset Computer Corporation, Bourne, MA, USA) mounted on the south-facing exterior wall of the building. A remote monitoring station datalogger (RX3000, Onset Computer Corporation, Bourne, MA, USA) interfaced ambient sensors and the four wireless temperature/relative humidity sensors (±0.20°C from 0 to 70°C, ± 2.5% from 10 to 90% RH RXW-THC-B, Onset Computer Corporation, Bourne, MA, USA) placed inside the test section. Data were recorded at 15-min intervals and automatically uploaded to the cloud *via* an onboard cellular gateway.

Two temperature sensors (±0.2°C from 0 to 70°C; MX2304, Onset Computer Corporation, Bourne, MA, USA) were suspended in the headspace (i.e., between manure level and slats). Additionally, one temperature sensor was submerged approximately 0.5 m into the manure. Slat temperature was recorded with a data logger (UX120-014M, Onset Computer Corporation, Bourne, MA, USA) connected to thermocouples (Type J) embedded in the concrete and sealed in place with a flexible epoxy.

The amount of propane used to heat the room was measured with a gas meter (PGM-075, EKM Metering Inc., Santa Cruz, CA, USA) connected inline between the tank and the heater. The gas meter output was connected to a 4-channel pulse datalogger (UX120-017M; Onset Computer Corporation, Bourne, MA, USA). An independent alarm system (BarnTalks, BarnTools LLC, Clive, IA, USA) was installed as a backup to ensure room conditions were maintained and to alert personnel if the temperature exceeded a threshold of ±3°C of the set point.

### Carcass management

A total of 18 swine carcasses were supplied by a local cooperator (euthanized prior to procurement). The carcasses were divided into three weight ranges (feeder, market, and sow) and weighed upon arrival at the facility. Scale accuracy was verified with seven 50 lb traceable weights. Three carcasses from each weight range were randomly allocated to one of two treatment groups: (1) carcasses placed directly on slats (SLATS) or (2) carcasses placed on a raised platform (RAISED). The locations of the treatment groups and carcass weights were randomly allocated within the room. For carcasses placed directly on the slatted concrete flooring (SLATS), any leachate or fluids emerging from the carcasses were allowed to disperse and drain into the manure pit below. The RAISED treatment group consisted of carcasses placed on a 0.5 (W) by 2 (L) by 0.2 (H) m grated iron platform on top of a waterproof tarp, with its perimeter wrapped around a set of three foam pool noodles to form a collection vessel for any leachate percolating from the decaying carcass. The carcasses in each room and the leachate from the RAISED treatment group were weighed using the same scale on completion of the trial.

The carcasses were placed and instrumented inside the test section on the morning of May 25, 2021. The set point was configured to 54.4°C (130°F) and the heat was turned on at 12:00. The room temperature was held constant for 14 days and the carcasses were then removed, beginning at 08:00 on June 9, 2021. Once removed, the carcasses were weighed and placed in a compost pile to complete decomposition.

### Carcass temperature

One carcass from each weight range and treatment group was randomly selected to be instrumented with six temperature sensors (±0.2°C from 0 to 70°C; MX2303, Onset Computer Corporation, Bourne, MA, USA). Five temperature sensors were inserted into various locations inside the carcass and the remaining sensor was attached directly to the skin surface, to represent local conditions. Data were collected at 15-min intervals. The five inserted sensor locations were: the brain, pleura, peritoneum, ham, and the medullary cavity of the femur. A portable drill with a 3/8-inch diameter bit was used to make a hole in the skull, approximately 4 cm deep, to access the cranial tissue. A 6-inch posting knife was used to make an incision to gain access to either the left or right stifle joint. A hole was drilled through the trochlear groove of the femur into the medullary cavity. The probe was then passed into the medullary cavity until it could no longer be advanced. The plural and peritoneal regions were more readily accessible, and sensors were inserted into small cross-shaped incisions using a scalpel. The ham consisted of muscle and tissue and was accessed by cutting incrementally larger cross-shaped incisions using a scalpel. All sensors were adhered using a flexible epoxy and sealed with duct tape to reduce movement throughout the experiment.

### Data and statistical analysis

All data were exported to a .csv file for pre-processing. Data were checked for errors, outliers, and completeness. For analysis, data were segregated into a transient and steady phase. The transient phase was defined as the period when the carcasses were heating. The steady-state phase was defined as when the carcass temperatures reached thermal equilibrium with the room environment (air, flooring, surroundings, etc.,), and any changes were attributed to changes in ambient conditions. In addition, due to the incomplete full days on the placement and removal of carcasses, data before day 0 (May 25, 2021) and after day 14 (June 9, 2021) were discarded.

A Linear Mixed Model was used in R (RStudio Team, 2020) to analyze the relationship between local carcass external temperature and the five temperature measurement locations inside the carcasses. Fixed effects included external temperature, measurement location, and the interaction term between the external temperature and measurement location. Random effects included the interaction between day and measurement location, where the intercept was set at each term's air temperature. Estimated marginal means were predicted at external temperatures of 40.6°C (105°F), 43.4°C (110°F), and 46.2°C (115°F) to compare the means of each group. A pairwise test was then conducted on the marginal means to obtain statistical results, with *P* ≤ 0.05 indicating significance and 0.05 < *P* ≤ 0.10 considered a trend for significance.

A Linear Mixed Model was used to analyze the relationship between internal carcasses temperatures for carcasses placed on slats (SLATS) compared to carcasses placed on the raised platform (RAISED). Fixed effects were an interaction effect between the temperature at each measurement location (i.e., brain, pleura, peritoneal, ham, and bone marrow of femur), weight group, and placement, and three two-way interactions between air and body part temperature, weight group, and group. Random effects included an interaction between day, measurement location, and carcass identification number, where each intercept was set at each term's designated air temperature. Estimated marginal means were predicted at skin/air temperatures of 40.6°C (105°F), 43.4°C (110°F), and 46.2°C (115°F) to compare the differences between body parts at these temperatures. Statistical results with P ≤ 0.05 indicating significance and 0.05 < *P* ≤ 0.10 considered a trend for significance.

A Univariate model was used to analyze the effects of carcass placement (i.e., SLATS and RAISED) on final carcass weight. Fixed effects included placement and weight group. A random effect was pig identification. Estimated marginal means were predicted between the carcasses in the SLATS and RAISED treatment groups.

## Results

**Figure 2:**
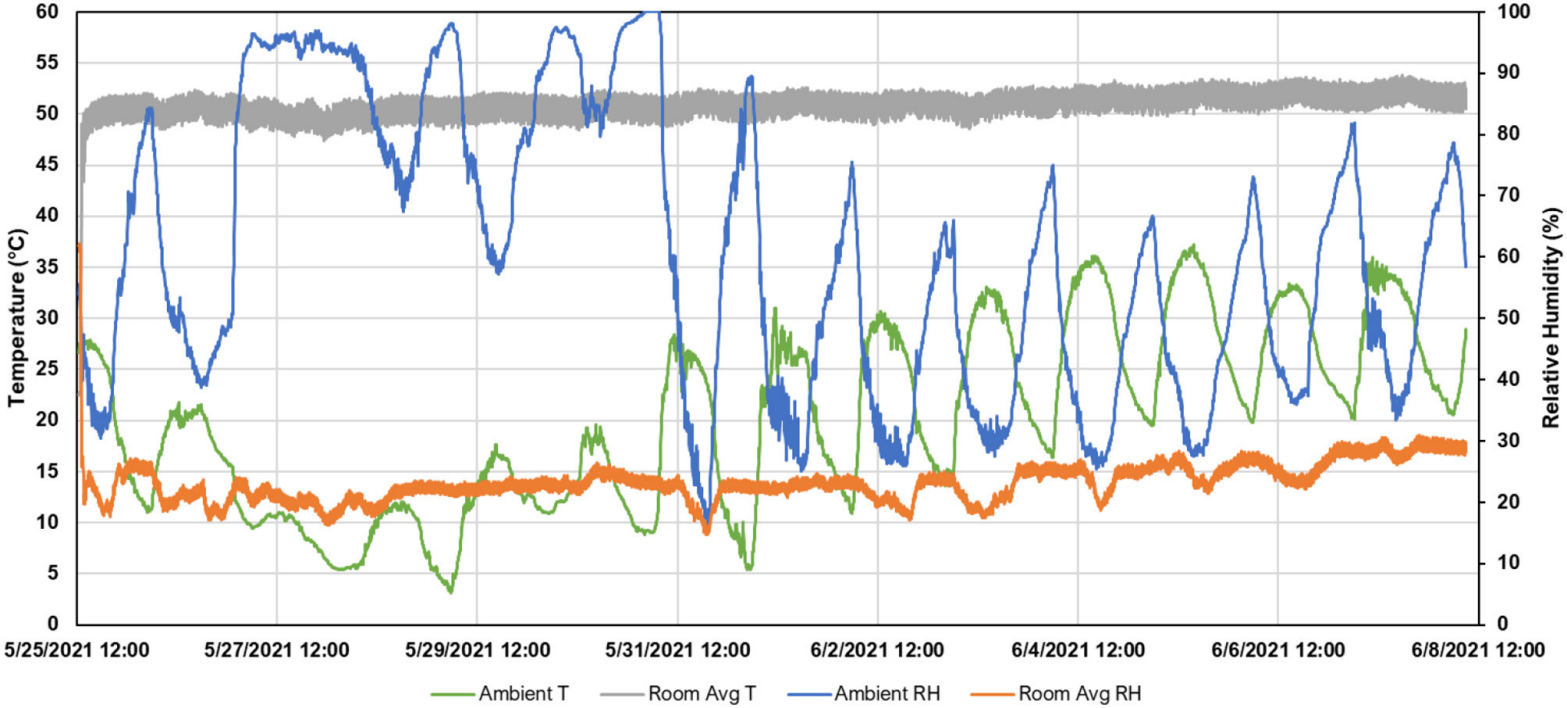
Summary of ambient and room conditions during the 14-day period.

Ambient (outdoor) and room conditions are presented in Figure 2. The average (±SD) ambient temperature was 22.6°C ± 8.8°C (68.2°F ± 15.8°F), with a minimum and maximum of 3.5°C (37.7°F) and 41.8°C (98.9°F), respectively. The average ambient relative humidity was 60.8% ± 23.0%, with a minimum and maximum of 15.4 and 100.0%, respectively. Room temperature reached its steady-state temperature in approximately 1.5 h, which was about 3.3°C (6°F) less than the setpoint temperature. Throughout the 14-day period, the average room temperature was 57.3°C ± 1.2°C (123.7°F ± 2.2°F), with a minimum and maximum of 53.3°C (117.3°F) and 60.5°C (128.8°F), respectively. The average room relative humidity was 23.1% ±3.3%, with a minimum and maximum of 14.6 and 62.2%, respectively.

**Figure 3:**
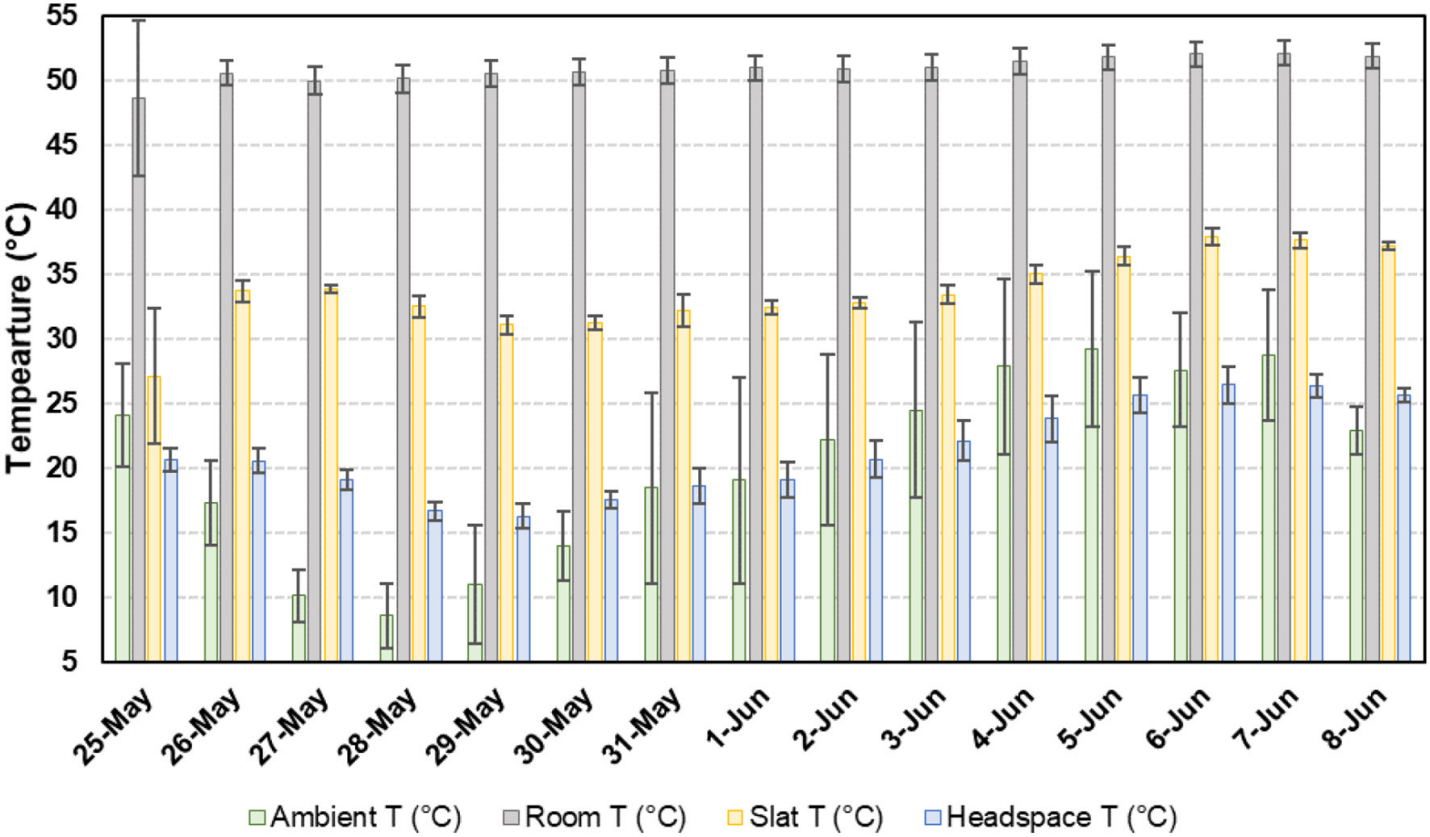
Average daily conditions for ambient (outdoor), room, slat, and headspace temperature (T).

**Figure 4:**
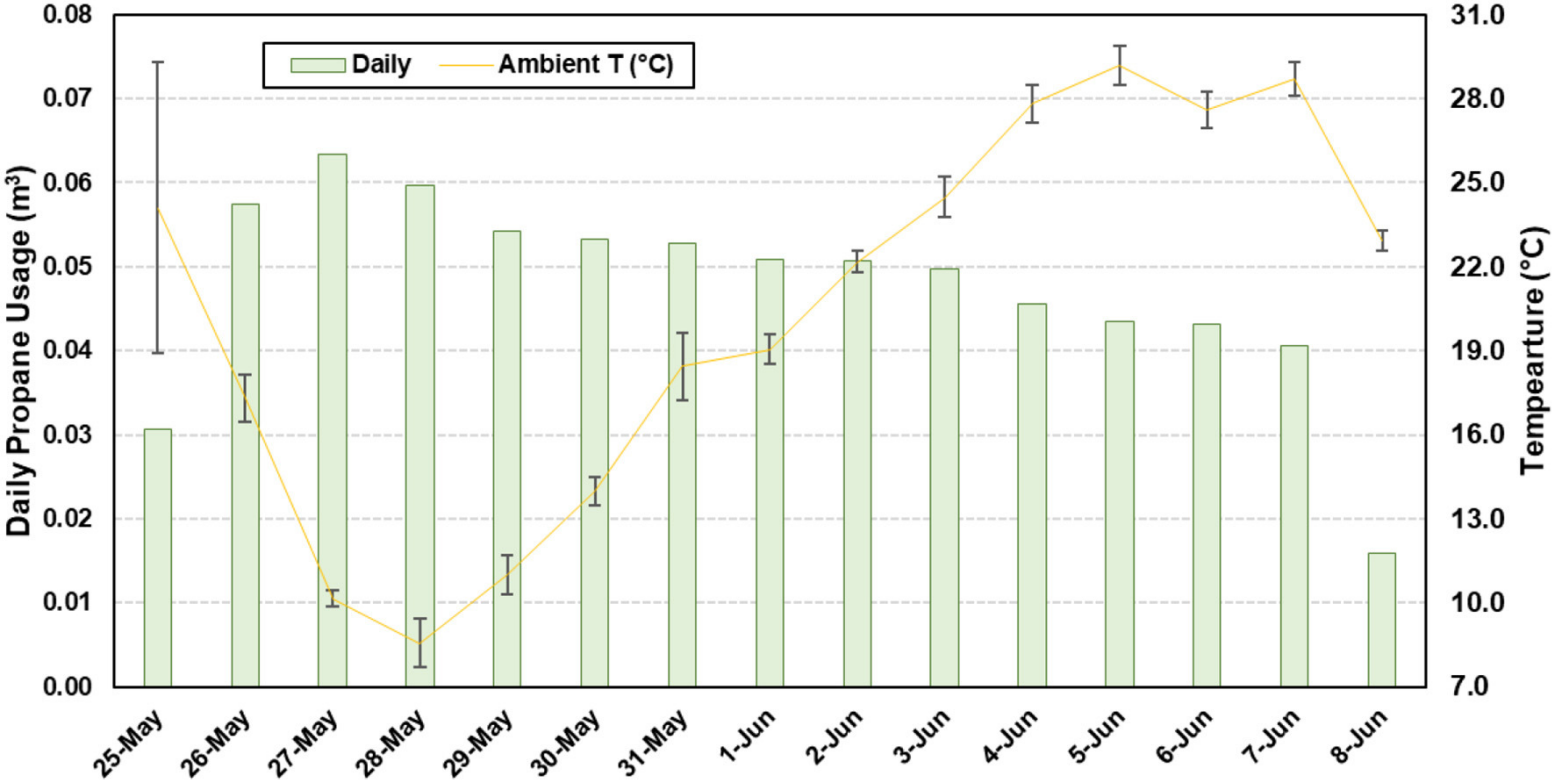
Daily propane usage superimposed over ambient temperature (T) to maintain the room temperature.

Temporal temperature data for the concrete slats and headspace are presented in Figure 3. The average slat temperature was 22.6°C ± 8.8°C (68.2°F ± 15.8°F), with a minimum and maximum of 3.5°C (37.7°F) and 41.8°C (98.9°F), respectively. The average headspace temperature was 22.6°C ±8.8°C (68.2°F ± 15.8°F), with a minimum and maximum of 3.5°C (37.7°F) and 41.8°C (98.9°F), respectively. The average slurry temperature was 22.6°C ± 8.8°C (68.2°F ± 15.8°F), with a minimum and maximum of 3.5°C (37.7°F) and 41.8°C (98.9°F), respectively. A total of 0.72 m^3^ (156.6 gallons) of propane was used to maintain the room temperature (Figure 4). As ambient air temperature increased, propane usage decreased.

### Carcass management

Initial and final carcass weights for each of the three weight groups as well as the collected leachate weights (RAISED only) are presented in Table 1.

**Table 1:** Average (SD) values by carcass weight group summarizing carcass weight loss and leachate weight collected for carcasses placed directly on slats (SLATS; no leachate collection) and raised above slats (RASIED).

	**Carcass weight group**	**Initial carcass weight (kg)**	**Final carcass weight (kg)**	**Leachate weight (kg)**	**Carcass weight loss (kg)**	**Carcass weight loss (kg/day)**	**Percent carcass weight loss**	**Leachate fraction of weight loss**
SLATS	Feeder	29.2 (0.43)	15.9 (1.56)	–	13.2 (1.21)	1.0 (0.09)	45.0% (4.70%)	–
	Market	97.2 (6.25)	64.7 (9.41)	–	32.5 (8.57)	2.3 (0.61)	33.0% (8.30%)	–
	Sow	208.7 (12.29)	138.2 (35.87)	–	70.5 (31.35)	5.0 (2.24)	34.0% (15.80%)	–
RAISED	Feeder	34.5 (2.67)	21.2 (3.89)	5.2 (2.30)	13.2 (1.37)	1.0 (0.10)	39.0% (6.80%)	39.0% (15.90%)
	Market	108.1 (5.77)	55.3 (11.56)	23.4 (9.57)	52.8 (14.10)	3.8 (1.01)	49.0% (11.30%)	44.0% (9.00%)
	Sow	244.0 (27.31)	135.8 (30.70)	55.8 (29.48)	108.2 (5.05)	7.7 (0.36)	45.0% (6.70%)	50.0% (24.30%)

Table 2 summarizes the statistical analysis, comparing carcass placement and carcass weight loss. For the feeder carcass weight group, there was no effect of placement on carcass weight loss. For the market weight group, there was an effect of carcass placement on carcass weight loss. For the sow weight group, there was a trend for significance for the effect of carcass placement on carcass weight loss. Because the interaction between placement and carcass weight group was significant, we chose to ignore the overall effects.

**Table 2:** Contrasts between the floor and raised carcasses by carcass weight group.

**Carcass weight group**	**Treatment group**	**Estimate (kg)**	**SE (kg)**	**Contrast (SLATS–RAISED) (kg)**	***P*-value of contrast**
Feeder	SLATS	−7.59	16.16	0.35	0.97
	RAISED	−7.94	15.29		
Market	SLATS	−31.31	6.36	21.02	0.03
	RAISED	−52.33	5.66		
Sow	SLATS	−98.67	17.77	18.03	0.13
	RAISED	−116.70	23.51		
Overall	SLATS	−45.0	3.51	13.10	0.04
	RAISED	−59.0	4.03		

Estimates are LS means carcass weight loss and the contrast shows the difference in estimates.

### Carcass temperature

**Figure 5:**
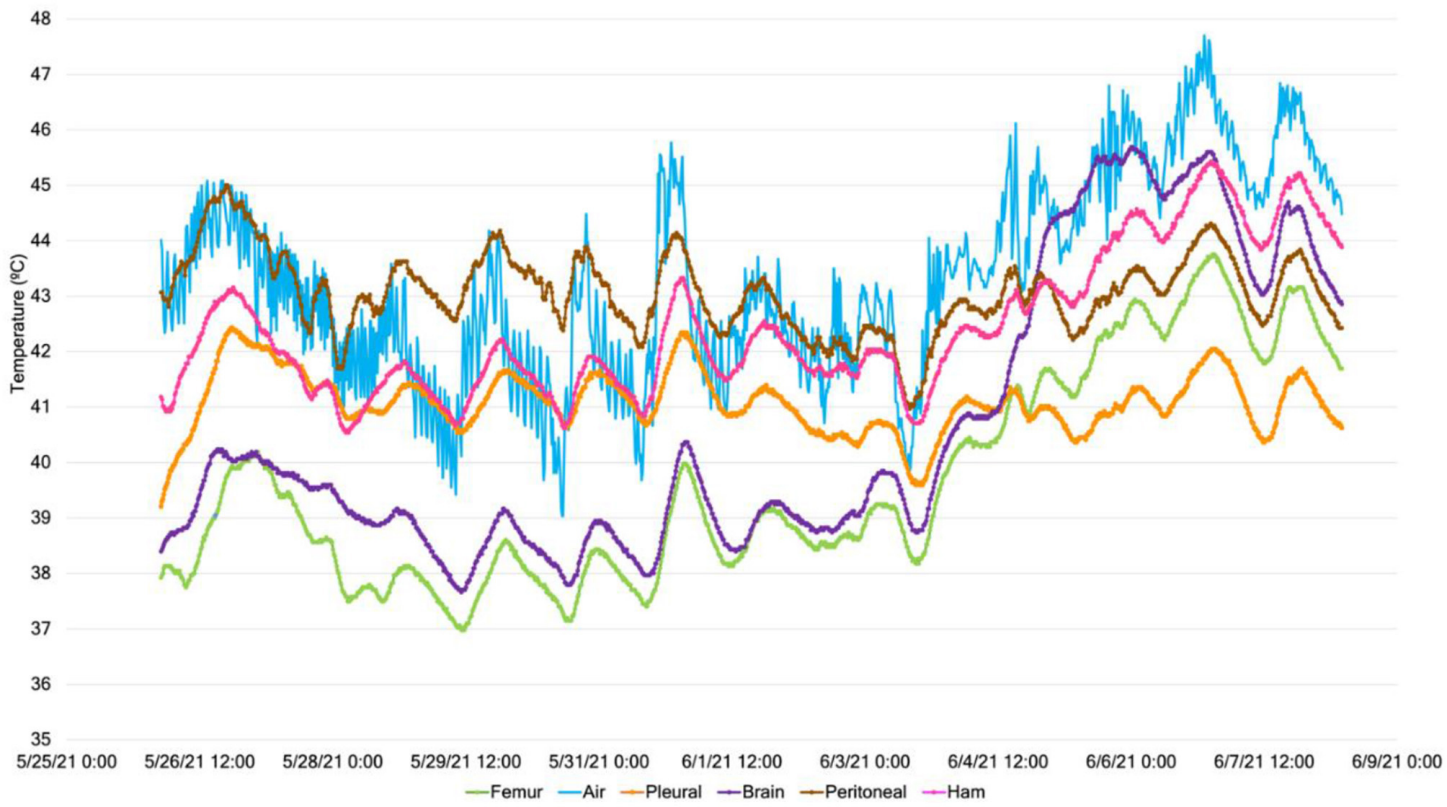
Example (carcass weight group: feeder; carcass ID #4) temperature data throughout the 14-day study at a constant air temperature of 50°C for the five internal carcass measurement locations and the external surface.

Of the 36 temperature sensors, two temperature channels failed to record data, resulting in no data. After 1 day, carcass temperatures reached steady-state conditions, as shown by the temporal data of an example feeder pig depicted in Figure 5. All internal measurement locations trended together in response to the ambient conditions.

The summary statistics for the six measured locations, averaged across carcass weight groups and placements, are presented in Table 3. For the feeder carcass weight group, the greatest temperature differences among measurement locations for the mean, minimum, and maximum temperatures were 5.7, 10.2, and 3.9°C, respectively. For the market carcass weight group, the greatest temperature differences among measurement locations for the mean, minimum, and maximum temperatures were 6.7, 8.4, and 5.4°C, respectively. For the sow carcass weight group, the greatest temperature differences among measurement locations for the mean, minimum, and maximum temperatures were 10.5, 13.7, and 7.2°C, respectively. This suggests that measurement location temperature differences showed increasing variation with increasing carcass weight.

**Table 3:** Summary statistics for the six measured locations averaged across carcass weight groups and treatment groups (SLATS and RAISED).

**Carcass weight group**	**Measurement location**	**Mean (SD) (°C)**	**Minimum (°C)**	**Maximum (°C)**
Feeder	Surface	41.6 (2.6)	36.0	47.7
	Brain	40.4 (2.3)	34.8	45.7
	Femur	36.9 (3.8)	29.9	43.8
	Pleural	38.5 (3.9)	30.9	44.5
	Peritoneal	42.6 (1.0)	40.1	45.0
	Ham	40.1 (3.2)	33.7	45.4
Market	Surface	43.4 (2.0)	38.5	47.6
	Brain	–^[a]^	– ^[a]^	– ^[a]^
	Femur	37.0 (3.1)	31.5	42.2
	Pleural	– ^[a]^	– ^[a]^	– ^[a]^
	Peritoneal	43.7 (1.5)	39.9	46.8
	Ham	43.1 (2.1)	39.3	46.4
Sow	Surface	46.5 (5.0)	41.9	51.0
	Brain	39.0 (1.8)	32.7	45.5
	Femur	36.0 (1.5)	28.9	43.8
	Pleural	44.3 (3.9)	39.9	46.2
	Peritoneal	45.9 (1.1)	42.6	48.0
	Ham	45.0 (1.5)	32.7	45.5

[a] Sensor malfunction caused data loss.

Table 4 shows the results of the statistical analysis for an example carcass, comparing the different internal measurement locations. These contrasts were created for each carcass and assessed. For the three temperature levels, results show that there were typically temperature differences between the brain and the ham and peritoneal, the femur and the ham and pleural, the ham and the peritoneal, and the peritoneal and pleural. Specifically, there were also temperature differences between the femur and pleural, at 40.5 and 43.3°C temperature levels.

**Table 4:** Contrasts of different internal measurement locations at the three internal temperature levels for an example carcass (carcass weight group: feeder; carcass ID #4).

**Temperature level**	**Measurement location contrast**	**Estimate (°C)**	**SE (°C)**	***P*-value of contrast**
40.5°C (105°F)	Brain–Femur	1.06	0.45	0.14
	Brain–Ham	−1.67	0.45	<0.01
	Brain–Peritoneal	−2.31	0.45	<0.01
	Brain–Pleural	−0.79	0.45	0.16
	Femur–Ham	−2.73	0.45	<0.01
	Femur–Peritoneal	−3.37	0.45	<0.01
	Femur–Pleural	−1.85	0.45	<0.01
	Ham–Peritoneal	−0.64	0.45	0.25
	Ham–Pleural	0.88	0.45	0.57
	Peritoneal–Pleural	1.52	0.45	<0.01
43.3°C (110°F)	Brain–Femur	1.00	0.44	0.23
	Brain–Ham	−1.78	0.44	<0.01
	Brain–Peritoneal	−2.42	0.44	<0.01
	Brain–Pleural	−0.44	0.44	0.43
	Femur–Ham	−2.79	0.44	<0.01
	Femur–Peritoneal	−3.42	0.44	<0.01
	Femur–Pleural	−1.44	0.44	<0.01
	Ham–Peritoneal	−0.63	0.44	0.25
	Ham–Pleural	1.35	0.44	0.12
	Peritoneal–Pleural	1.98	0.44	<0.01
46.1°C (115°F)	Brain–Femur	0.94	0.56	0.55
	Brain–Ham	−1.90	0.56	<0.01
	Brain–Peritoneal	−2.53	0.56	<0.01
	Brain–Pleural	−0.09	0.56	0.87
	Femur–Ham	−2.84	0.56	<0.01
	Femur–Peritoneal	−3.47	0.56	<0.01
	Femur–Pleural	−1.03	0.56	0.10
	Ham–Peritoneal	−0.63	0.56	0.45
	Ham–Pleural	1.82	0.56	0.06
	Peritoneal–Pleural	2.44	0.56	<0.01

To further assess the influence of carcass weight group on internal temperatures, Table 5 shows the results of the statistical analysis, comparing the marginal means difference for internal measurement locations by carcass weight group at each internal temperature level.

**Table 5:** Contrasts of temperature measurement locations between carcass weight groups for the three internal temperature levels.

**Temperature level**	**Measurement location**	**Carcass weight group contrast**	**Contrast (°C)**	**SE (°C)**	***P*–value of contrast**
40.5°C (105°F)	Brain	F–S	2.63	0.44	<0.01
	Femur	F–M	0.70	0.44	0.94
		F–S	1.82	0.44	0.08
		M–S	1.12	0.44	0.03
	Ham	F–M	−1.81	0.44	<0.01
		F–S	−3.47	0.44	<0.01
		M–S	−1.66	0.44	<0.01
	Peritoneal	F–M	−0.01	0.44	0.163
		F–S	−1.52	0.44	<0.01
		M–S	−1.51	0.44	<0.01
	Pleural	F–S	−4.46	0.44	<0.01
43.3°C (110°F)	Brain	F–S	6.57	0.44	<0.01
	Femur	F–M	0.65	0.44	0.85
		F–S	−0.63	0.44	0.004
		M–S	−1.27	0.44	0.02
	Ham	F–M	0.88	0.44	<0.01
		F–S	4.38	0.44	<0.01
		M–S	3.50	0.44	0.004
	Peritoneal	F–M	−1.59	0.44	0.61
		F–S	−2.84	0.44	<0.01
		M–S	−1.26	0.44	0.0101
	Pleural	F–S	−3.99	0.44	<0.01
46.1°C (115°F)	Brain	F–S	2.81	0.45	<0.01
	Femur	F–M	0.92	0.45	0.35
		F–S	2.00	0.45	<0.01
		M–S	1.08	0.45	0.02
	Ham	F–M	−1.59	0.45	0.001
		F–S	−3.29	0.45	<0.01
		M–S	−1.70	0.45	0.01
	Peritoneal	F–M	0.22	0.45	0.99
		F–S	−1.34	0.45	0.01
		M–S	−1.56	0.45	0.02
	Pleural	F–S	−4.29	0.45	<0.01

Carcass weight groups: *F*, feeder; M, market; S, sow.

Table 6 summarizes the statistical analysis to assess whether carcass placement (for all weight groups), on slats or raised, influenced internal temperature. At 40.5, 43.3, and 46.1°C, carcass placement had an effect on the temperature achieved in the femur and ham. The positive contrast indicates that the carcasses placed on the slats were colder than those raised for leachate collection. However, at those temperature levels, the peritoneal temperature was not affected by placement.

**Table 6:** Contrasts between carcass placement treatment groups for key internal temperature measurement locations, separated by three temperature levels.

**Temperature level**	**Measurement location**	**Contrast** **(RAISED–SLATS) (°C)**	**SE (°C)**	***P*–value of contrast**
40.5°C (105°F)	Femur	5.98	0.70	<0.01
	Ham	2.71	0.70	<0.01
	Peritoneal	−0.60	0.70	0.13
43.3°C (110°F)	Femur	5.93	0.69	<0.01
	Ham	2.65	0.69	<0.01
	Peritoneal	−0.65	0.69	0.09
46.1°C (115°F)	Femur	5.88	0.36	<0.01
	Ham	2.60	0.36	<0.01
	Peritoneal	−0.71	0.36	0.07

## Discussion

This novel study aims to characterize the environmental parameters during in-barn heat processing, understand the temperature dynamics inside swine carcasses of varying mass, and assess total carcass weight loss and degradation during heat processing.

### Carcass management

It was expected that the carcasses raised above the slats would lose more weight than those placed directly on the slats. The circulation of warm air both above and underneath the raised carcasses would increase the rate of drying, and subsequently, increase water loss. Numerically, the sow (*P* = 0.13) and market (*P* = 0.03) carcass weight groups in the raised treatment group showed greater average daily weight loss compared to those placed on the slats. However, only the market carcass weight group showed a statistical interaction, indicating that average market carcass weight loss was affected by placement. However, the feeder carcass weight group showed an average of 1.0 kg/day of weight loss for both the raised and slats treatments (*P* = 0.97). This may be attributed to the high surface area to mass ratio (8), which caused equal rates of desiccation. Information on carcass weight loss and leachate generation rates is valuable for evaluating morality management strategies.

### Carcass temperature

Considerable variations in carcass temperatures were recorded among different carcass weights and placements within the room. The results indicate that the internal temperature correlated with the ambient temperature. This is reasonable as the building envelope and unheated portion of the deep pit, while thermally massive, changed with ambient conditions, thus causing the carcasses to lose heat *via* conduction into the concrete slats. There was also infiltration of colder ambient air due to the large temperature difference between the room and outside. This thermal gradient drives greater air exchange and could lead to temperatures being influenced by outdoor temperatures in the headspace of the pit, which would affect the concrete slat temperature.

Internal carcass temperatures did not achieve the published time-temperature criteria for the thermal inactivation of the ASF virus (i.e., 56°C for 70 min and 60°C for 20 min); however, the average temperature over the 14-day period for all internal measurement locations was 41°C (SD = 3.2°C). The combination of time and temperature affects the degree of pathogen reduction achieved during heat treatment. That is, shorter exposure times at higher temperatures achieve levels of pathogen reduction that are analogous to those of longer exposure times at lower temperatures. While values for lower temperatures and longer exposure times are not currently available for ASF, Espinosa et al. (9) conducted a review to create zones of time-temperature combinations for common, food-related microbial groups (e.g., Salmonella, bacteriophage, enteric viruses, and Listeria). They showed that a 1-log_10_ (90%) reduction could be achieved for these microbial groups when exposed to temperatures >10°C and < 40°C for a duration of between 1 day and up to 6 months. More research into combinations of time and temperature of thermal inactivation for ASF is needed in order to allow for more disposal options in an ASF outbreak.

### Practical considerations

In response to a FAD outbreak, a heat treatment process applied inside a swine facility could limit virus spread through the thermal inactivation of the virus inside carcasses and building materials and reduce the weight of carcasses for disposal. Late into the 2014–2015 highly pathogenic avian influenza outbreak, the feasibility and cost-effectiveness of using heat treatment was demonstrated for virus elimination (in conjunction with cleaning). The developed procedure stated that barns/houses must be heated to between 37.7°C (100°F) and 48.9°C (120°F) for a total of 7 days, with at least three consecutive days (of the 7 days) of heating continuously to within this temperature range (6, 7). A thorough evaluation of the heat treatment process specific to swine facilities is needed due to the major differences between swine and poultry housing styles.

There are several potential advantages of heat-treating swine carcasses inside a facility. The pathogen degradation inside carcasses and facilities can be accelerated with heat to reduce the load inside the barn environment. This may help reduce the risk of transmission into the environment *via* land or water, or *via* scavenging vectors (e.g., coyote, bird, skunk, etc.) that can access outdoor mortality management approaches.

Carcass removal is the greatest challenge associated with this approach because most swine facilities are constructed with concrete slatted floors supported by columns and beams. This limits the possibility of using heavy, off-road equipment to remove carcasses. Without a mechanical method for carcass removal, the physically demanding labor required to remove potentially >150,000 kg of carcass is not practical. Furthermore, the heating process degrades soft tissue, rendering the carcasses more difficult to move. More work is needed on this topic to promote efficient carcass removal.

## Data availability statement

The raw data supporting the conclusions of this article will be made available by the authors, without undue reservation.

## Author contributions

BR: conceptualization, methodology, formal analysis, writing—original draft, funding acquisition, and project administration. RJ: formal analysis and writing—original draft. DS, KK, JB, and DM: conceptualization, investigation, and writing—review and editing. CR: conceptualization writing—review and editing. All authors contributed to the article and approved the submitted version.
